# Investigating sarcopenia and mucus plugging by chest computed tomography in patients with severe chronic obstructive pulmonary disease

**DOI:** 10.1038/s41598-026-49060-7

**Published:** 2026-04-28

**Authors:** Antonia Petersen, Ralf-Harto Hübner, Marcus A. Mall, Ingo G. Steffen, Oliver Weinheimer, Martin Witzenrath, Jacopo Saccomanno, Thomas Elgeti

**Affiliations:** 1 Department of Radiology, Charité – Universitätsmedizin Berlin, Corporate Member of Freie Universität Berlin and Humboldt-Universität zu Berlin, Hindenburgdamm 30, 12203 Berlin, Germany; 2 Department of Respiratory and Critical Care Medicine, Charité – Universitätsmedizin Berlin, Corporate Member of Freie Universität Berlin and Humboldt-Universität zu Berlin, Berlin, Germany; 3 Department of Pediatric Respiratory Medicine, Immunology and Critical Care Medicine, Charité – Universitätsmedizin Berlin, Corporate Member of Freie Universität Berlin and Humboldt-Universität zu Berlin, Berlin, Germany; 4 Department of Diagnostic and Interventional Radiology, University Hospital of Heidelberg, Heidelberg, Germany; 5 Translational Lung Research Center Heidelberg, German Center for Lung Research DZL, Heidelberg, Germany

**Keywords:** COPD, Mucus plugging, Sarcopenia, Muscle cross-sectional area, Anatomy, Diseases, Medical research

## Abstract

To determine the relationship between mucus plugging and CT-derived parameters of sarcopenia in routine chest CT-scans. Patients with advanced Chronic Obstructive Lung Disease (COPD GOLD 3 or 4) were investigated. Mucus plug score (MPS) and cross-sectional muscle area (CSA) of pectoralis and erector spinae muscle of each patient was assessed by two radiologists. Statistics included non-parametric group comparison, multivariate analysis, and inter- and intrarater agreement. Median age of 123 patients (47 female) was 66 years. In 63 patients (15 females) no mucus plugging was found. 31 patients (15 females) had 1–2 mucus plugs and 29 patients (17 females) had a mucus plug of ≥ 3. PM_CSA_ and ESM_CSA_ were not independently associated with MPS; however, the association between PM_CSA_ and MPS was modified by body weight, with a significant negative correlation between body weight and PM_CSA_ in patients with higher MPS (≥ 3). Inter- and intrarater agreement was very good (ICC 0.899 or higher). Imaging based evaluation of MPS and CSA is reliable on routine chest CT-scans. Patients with more advanced COPD exhibited a higher MPS and larger PM_CSA_ relative to body weight, possibly due to the greater muscular effort required for breathing.

## Introduction

Chronic obstructive pulmonary disease (COPD) is a leading cause of morbidity and mortality worldwide with exposition to cigarette smoke and air pollutants being the most important risk factors in combination with other genetic, environmental and social factors^1^. COPD is a heterogeneous respiratory condition characterized by airway and lung parenchyma remodeling with airway mucus obstruction being a key feature^2,3^. Mucus plugging (MP), i.e. the accumulation of mucus in the airways as plugs, is associated with increased mortality in patients with COPD and can be detected and quantified with chest computed tomography (CT)^4,5^. Imaging is a field of intensive research in patients with COPD^6^.

COPD is associated with numerous systemic manifestations and comorbidities, including sarcopenia^7^. Sarcopenia can be defined as low muscle strength combined with a decline in muscle quantity or quality^8,9^. Sarcopenia has shown to be an important factor influencing the outcome of the ageing population in different clinical diseases^10,11^. Patients with COPD and sarcopenia are at increased risk of adverse outcomes, including increased mortality^12,13^. Diagnosis and treatment of sarcopenia in COPD is essential as optimal care prevents high personal, social and economic burden^8^. Recently, imaging-based parameters of sarcopenia measured on CT have been introduced, e.g. cross-sectional area (CSA) of the pectoralis (PM) and erector spinae muscle (ESM) and muscle-density (MD)^14–18^. These measures have shown associations to various diseases^19,20^ and are currently field of extensive research^21–23^.

However, it is unknown if patients with advanced COPD and MP have altered imaging-based parameters of sarcopenia. The aim of this study was therefore to assess patients with advanced COPD for the presence of MP and its relationship with muscle CSA and muscle composition on thoracic CT scans. Our hypotheses were as follows: first, muscle CSA can reliably be assessed on thoracic CT scans of patients with advanced COPD. Second, MP might alter muscle CSA and composition.

## Materials and methods

### Study population

**Fig. 1:**
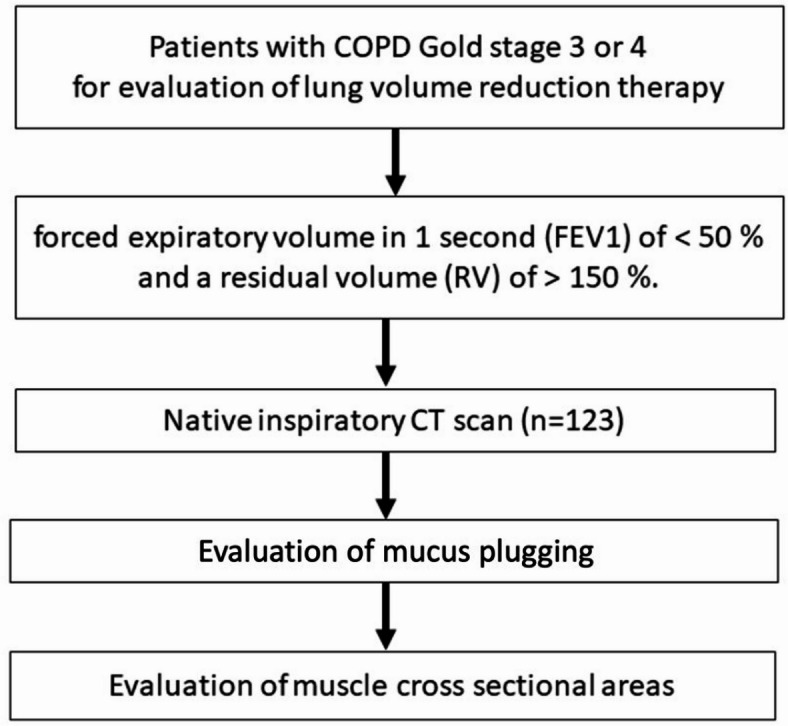
Flowchart showing the patient selection, imaging and performed analyses.

This is an internal review board-approved monocentric study (Ethikkommission der Charité—Universitätsmedizin Berlin, Charitéplatz 1, 10,117 Berlin, initial approval by the ethics committee no. EA2/149/17 and amendment no. EA2/149/23, German Clinical Trials Register no. DRKS00021207), that conforms to the declaration of Helsinki. All patients gave written informed consent. In total 123 consecutive patients (47 female) with COPD GOLD stage 3 or 4 disease who had undergone native chest CT to evaluate lung volume reduction therapy between 2017 and 2023 were included. Our patient cohort was part of previously reported data^24,25^. Patients were assessed according to the standards of the lung emphysema registry (www.lungenemphysemregister.de). Inclusion and exclusion criteria were applied as previously described^25–27^. Inclusion criteria included a forced expiratory volume in 1 s (FEV1) of < 50% and a residual volume (RV) of > 150% (Fig. 1). All patients were required to be non-smokers for at least three months. Patients’ symptoms had to be primarily attributed to lung emphysema, with dyspnea as the lead symptom and without chronic cough and sputum production. The demographic and clinical patients’ characteristics were obtained from the patients’ records and via REDCap electronic data capture tools managed by CAPNetz^28^.

### Chest computed tomography

Native inspiratory chest CT images were acquired on different CT scanners. Typical acquisition parameters were: 320-detector row/64-detector row/8-detector row CT scanner, tube voltage 120 kVp/120 kVp/120 kVp, reference tube current–time product 120 mAs/100 mAs/100 mAs with tube current modulation, rotation time 0.5 s/0.35 s/0.5 s; collimation 0.5 × 80 mm/0.625 × 40 mm/1.25 × 10 mm, pitch 1.813/1.374/1.675). The images were first reconstructed using a soft tissue kernel with a field of view of 512 × 512 mm each and an effective slice thickness of 1 mm/0.625 mm/1 mm. For assessment of the mucus plug score (MPS) the clinical imaging viewer´s multiplanar reconstructions (MPR)-tool (Visage® 7 version 7.2., Visage Imaging GmbH Berlin, Germany) and the lung window with a window level of -550 HU and a window width of 1600 HU was used.

### Assessment of the mucus plug score

**Fig. 2:**
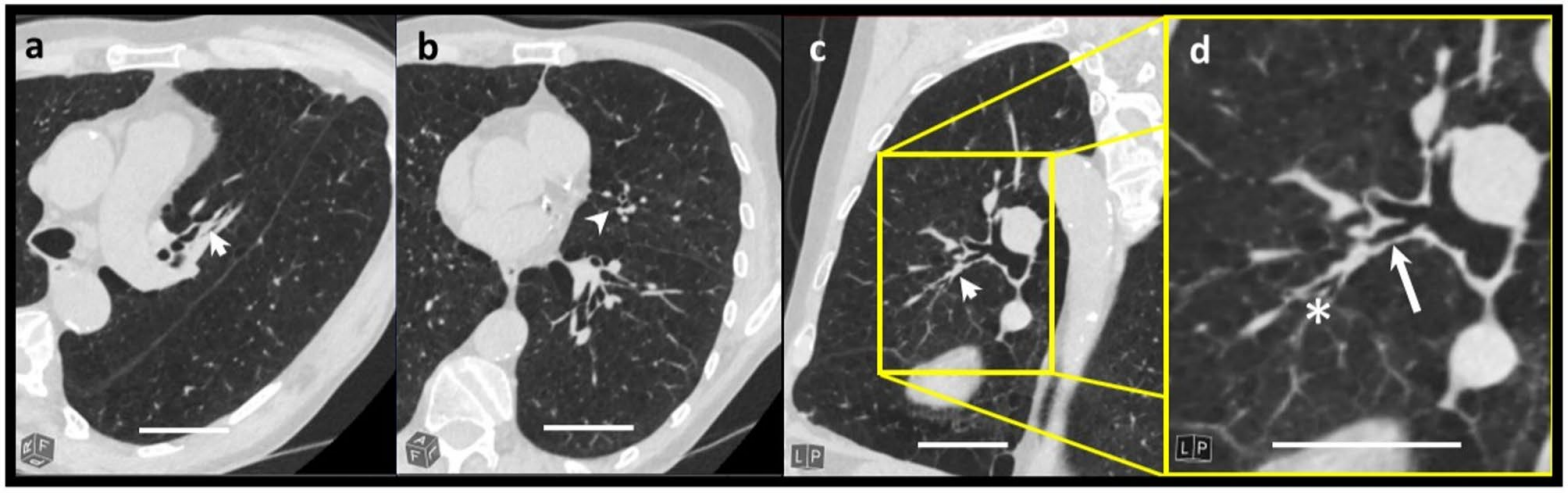
Example of a mucus plug in a 59-year-old male with COPD stage 4. The thin slice multiplanar reconstruction (MPR) shows segmental bronchial obstruction of the inferior lingula segment (arrowhead in **a**, **b**, **c**). **d** shows an image section of c. An orientation cube is displayed in the left lower corner of each image. The length of 50 mm is given by the white bar. In the right part of the figure the air filled proximal (arrow) and distal bronchial structures (asterix) can be easily identified on the magnified image in the yellow frame.

The MPS was assessed visually by two radiologists (A.P. with 5 and T.E. with > 15 years of experience in thoracic imaging) using the 20 point score as proposed by Dunican^29^: A mucus plug was defined as a focal complete obstruction of an airway with mucus, which appeared as a tubular or round opacification, depending on whether the bronchus was displayed in longitudinal or cross-sectional orientation. Airways within the 20 mm peripheral zone or airways that were only partially occluded were excluded. Maximum intensity projection (MIP) reconstructions were used to determine the distance of a mucus plug from the pleura as accurately as possible. One point was awarded for each bronchopulmonary segment in which MP was found. The segment scores were summed to generate the MPS. The patients were then categorized according to the MPS and divided into three groups: MPS equal to 0, MPS 1 or 2 and MPS greater than or equal to 3^5^. Interrater agreement was calculated based on the metric mucus plug (MP) evaluations from both readers. In order to determine the intrarater agreement, one rater (T.E.) repeated part of the evaluation (2nd rating, n = 70). Figure 1 gives an overview over the patient selection, imaging and performed analyses. An example of a mucus plug is displayed in Fig. 2.

### Assessment of muscle CSA and MD

**Fig. 3:**
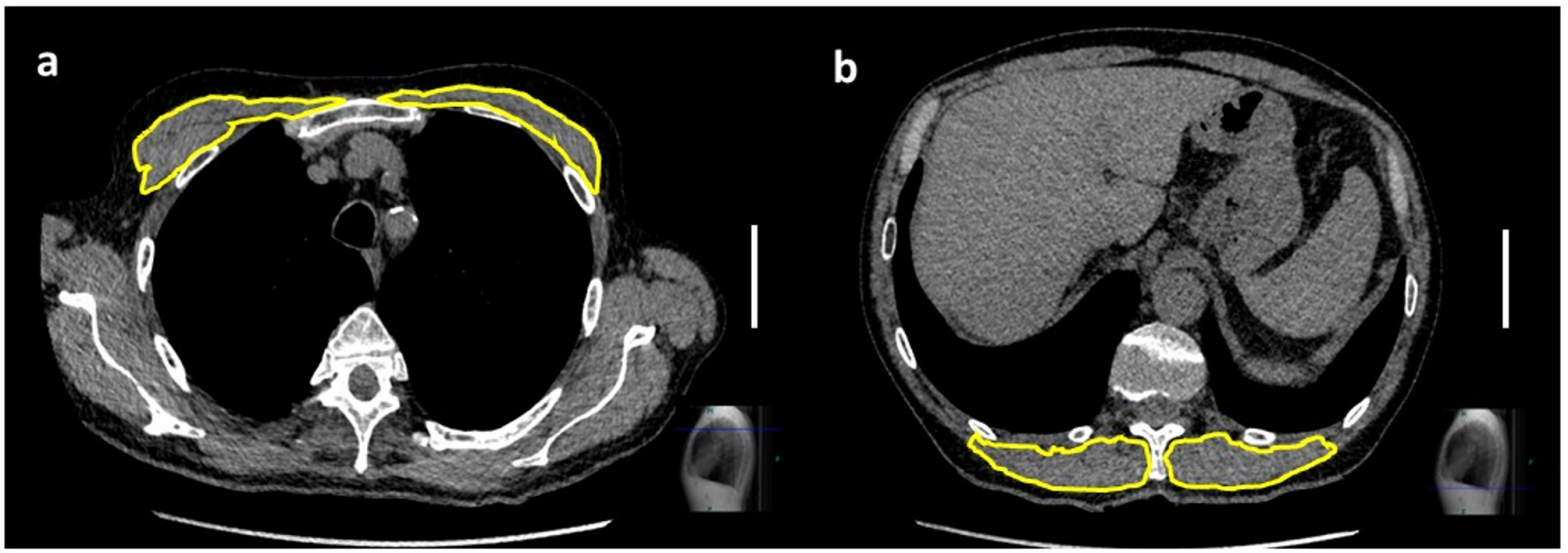
Example of the quantification of muscle area in transverse chest CT scans. (**a**) Both pectoralis muscles cross sectional areas (CSA) at the level of the aortic vascular outlets are demarked by a yellow line. (**b**) Delineated erector spinae muscle on the level of the thoracolumbar junction (Th12/L1). The pectoral muscles can be quantified with 15.70 cm^2^ and 11.10 cm^2^ and a mean density of 37.00 HU and 33.20 HU on the right / left side respectively. For the erector spinae the muscle measures 17.00 cm^2^ and 15.20 cm^2^ with a mean density of 32.00 and 41.60 HU on the right / left side respectively. The length of 50 mm is given by the white bar. The slice level is given by a blue line in the lateral scout view displayed in the left part of the image.

CSA and MD of the PM and the ESM were measured similarly to methods published previously^14,15,30^. Shortly, semi-automatically a contour was drawn around the corresponding muscle outline using the clinical imaging viewer´s ROI tool (an example is given in Fig. 3); the contours were corrected manually if necessary. PM_CSA_ and PM_MD_ were determined on the height on the first axial slice above the aortic arch, ESM_CSA_ and ESM_MD_ on the height of the base plate of the 12th thoracic vertebra. For the assessment of interrater agreement of CSA and metric MPS, 20 randomly selected patients were evaluated by the second reader (A.P.). To calculate intrarater agreement, the first reader re-evaluated these patients after nine months (T.E.).

### Statistical analysis

The software R® 4.4.2 (R Core Team (2021), R: A language and environment for statistical computing, R Foundation for Statistical Computing, Vienna, Austria) and IBM SPSS Statistics® 28.0.1.0 (IBM Corporation, Armonk, New York, United States) were used for statistical analysis performed by I.G.S. and A.P.. Descriptive parameters are given as median and inter-quartile-range (IQR) and samples are depicted as scatterplots for three different MPS groups, for which Spearman correlation coefficient (*ρ*) was calculated. The group comparison for all metric parameters was carried out using the Kruskal–Wallis-test, followed by a Mann–Whitney-U test with Bonferoni-Holm adjustment. A chi-square test was conducted to compare binarized variable gender and MPS. Inter- and intrarater agreement for analysis of metric MPS and sarcopenia parameters was determined using the intraclass correlation coefficient (two-way random, absolute agreement resp. one way random). Linear regression analysis was used to test for possible effects of age, gender, body weight, FEV1, PM_CSA_, ESM_CSA_ on metric MPS, followed by a multivariate analysis. The Akaike information criteria algorithm was used to use the final best model. To account for the significant differences in body weight between the different MPS groups, the correlation between PM_CSA_ and ESM_CSA_ and body weight was analyzed separately in each MPS group^31,32^. A linear regression model with an interaction term (body weight, PM_CSA_) was used to assess associations with the MPS and to test whether the association between PM_CSA_ and MPS varied by body weight. A p-value ≤ 0.05 was considered statistically significant.

## Results

### Demographics and clinical patients’ characteristics

All 123 patients (47 female) had COPD stage 3 or 4 disease. Median age was 66 years (IQR 9 years), mean body weight 67 kg (ICR 26 kg) and mean body height 170 cm (ICR 13 cm). Median FEV1 of all patients was 0.68 l (IQR 0.31 l), median VC was 2.08 l (IQR 1.00 l), median RV was 5.03 l (IQR 1.69 l) and partial pressure of carbon dioxide (pCO2) was 39.00 mmHg (IQR 7.75 mmHg).

### Analysis of MP and CT-derived parameters of sarcopenia

**Fig. 4:**
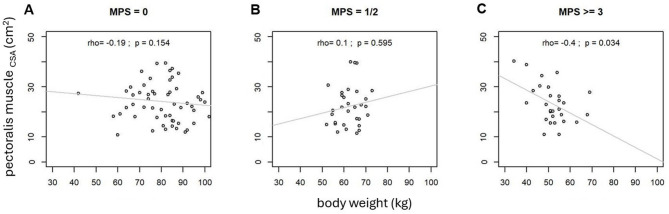
Scatterplots showing PM_CSA_ and body weight for the three different groups of mucus plugging: No mucus plugging (MPS = 0) on the left side (**A**), a MPS of 1 and 2 in the middle (**B**) and MPS of 3 and more than 3 on the right side (**C**). Each point represents an individual measurement; grey lines indicate the linear regression within each group. Reported values correspond to the Spearman correlation coefficient (ρ) and the associated p-value for each group. No significant correlation between body weight and PM_CSA_ can be found for mucus plugs 0–2 (**A**, **B**). In patients with MPS ≥ 3 (**C**), however, a moderate negative correlation is evident, indicating that higher body weight can be associated with lower PM_CSA_.

No MP was found in 63 (15 female) patients. 31 (15 female) patients had a MPS of at most 2, 29 patients (17 female) had a MPS of at least 3. There were no significant group differences with regard to age (*p* = 0.420) nor in gender ratio in patients with MP (*p* = 0.427). Patients with a higher MPS had a significantly lower body weight (*p* < 0.001) with significant differences between all groups (MPS 0 vs. MPS 1, 2, *p* < 0.001; MPS 0 vs. MPS ≥ 3, *p* < 0.001; MPS 1,2 vs. MPS ≥ 3, *p* = 0.005). There were significant differences in FEV1 with a higher MPS being associated with lower FEV1 values (*p* < 0.001) with a significant difference between each group except between patients with an MPS of at most 2 and an MPS of at least 3 (MPS 0 vs. MPS 1, 2, *p* = 0.007; MPS 0 vs. MPS ≥ 3, *p* < 0.001; MPS 1, 2 vs. MPS ≥ 3, *p* = 1.000). These findings are summarized in Table 1. There were no significant differences between the MPS groups in terms of PM_CSA_ and ESM_CSA_ (*p* = 0.764 resp. 0.690) nor in terms of PM_MD_ and ESM_MD_ (*p* = 0.283 resp. *p* = 0.688). These findings are summarized in Table 2. Scatterplots are used to display the association of PM_CSA_ and body weight for the three different MPS groups in Fig. 4. Here the groups with MPS of at least three show a moderate significant negative correlation with *ρ* =  − 0.4 (*p* = 0.003).

**Table 1:** Overview over the demographic and clinical data categorized according to the mucus plug score. MPS, mucus plug score; FEV1, forced expiratory volume in one second. Values are presented as median (interquartile range). The *p*-values show the result of the group comparison using the Kruskal–Wallis-test.

MPS	0	1, 2	≥ 3	*p*-value
n (female)	63 (15)	31 (15)	29 (17)	** < 0.001**
Age (years)	66 (9)	66 (7)	65 (9)	0.420
Body weight (kg)	82.00 (16.00)	62.00 (8.00)	51.50 (6.75)	** < 0.001**
FEV1 (l)	0.80 (0.38)	0.64 (0.18)	0.61 (0.23)	** < 0.001**

A *p*-value of < 0.05 was considered statistically significant.

**Table 2:** Results of the analysis of CT-derived parameters of sarcopenia categorized according to the mucus plug score.

MPS	0	1, 2	≥ 3	*p*-value
PM_CSA_ (cm^2^)	21.80 (11.55)	20.18 (11.58)	20.71 (9.91)	0.764
ESM_CSA_ (cm^2^)	32.50 (13.70)	31.00 (6.00)	30.55 (14.67)	0.690
PM_MD_ (HU)	30.25 (16.48)	33.50 (13.05)	33.90 (15.00)	0.283
ESM_MD_ (HU)	36.05 (13.20)	35.85 (16.80)	37.55 (12.75)	0.688

MPS, mucus plug score; PM, pectoralis muscle; ESM, erector spinae muscle; CSA, cross sectional area; MD, muscle density; HU, Hounsfield Units.

Values are presented as median (interquartile range). The *p*-values show the result of the group comparison using the Kruskal–Wallis-test.

### Multivariate analysis

A linear regression was performed to assess the relationship between age, gender, body weight, PM_CSA_, ESM_CSA_, FEV1. Age did not significantly predict MPS (R^2^ = 0.001, *p* = 0.716), while gender was a significant predictor of MPS (R^2^ = 0.081, *p* = 0.001). PM_CSA_ did not significantly predict MPS (R^2^ = 0.005, *p* = 0.464), as well as ESM_CSA_ (R^2^ = 0.001, *p* = 0.780). Body weight was a significant negative predictor of MPS (R^2^ = 0.336, *p* < 0.001), the same was true for FEV1 (R^2^ = 0.127, *p* < 0.001). In a multivariable model, that included age, gender, body weight, PM_CSA_ and FEV1, body weight and FEV1 were significant negative predictors of MPS (adjusted R^2^ = 0.344, *p* < 0.001), whereas age, gender, PM_CSA_ were not significant. The final Akaike information-optimized model included body weight and FEV1 (lowest AIC 461.94; adjusted R^2^ = 0.354).

### Analysis of association MP, body weight and CT-derived parameters of sarcopenia

**Fig. 5:**
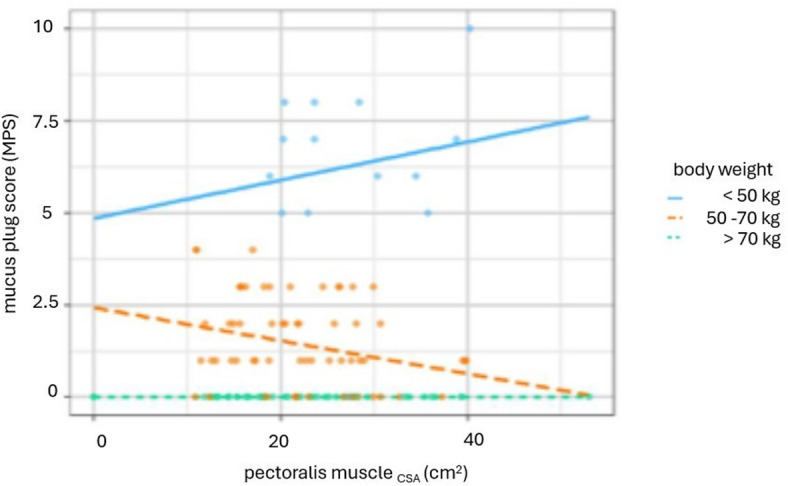
Interaction plot showing the relationship between mucus plug score (MPS), cross-sectional area of the pectoralis muscle (PM_CSA_) and body weight for patients with a body weight below 50 kg, a body weight between 50 and 70 kg and a body weight higher than 70 kg. In our patient group, these categories correspond to BMI of less than 18.5 (underweight), 18.5–24.9 (normal weight) and greater than 25 (overweight).

Patients with no MP (MPS = 0) showed no significant correlation between body weight and PM_CSA_ and ESM_CSA_ (ρ =  − 0.19 resp. ρ =  − 0.04, *p* > 0.05). There was also no significant correlation between body weight and PM_CSA_ resp. ESM_CSA_ in the group of patients with an MPS of at most 2 (MPS = 1,2) with ρ = 0.10 resp. ρ = 0.14 (*p* > 0.05). Patients with an MPS of at least 3 (MPS ≥ 3) showed a significant negative correlation between body weight and PM_CSA_ with ρ =  − 0.40 (*p* ≤ 0.05), but no significant correlation in terms of body weight and ESM_CSA_ (ρ =  − 0.16). In a linear regression analysis, MPS was significantly associated with body weight, PM_CSA_, and the interaction between body weight and PM_CSA_ indicating that the relationship between PM_CSA_ and body weight varies across levels of MPS, such that higher MPS is associated with higher PM_CSA_ at lower weight, whereas lower MPS is associated with lower PM_CSA_ at higher body weight (adjusted R^2^ = 0.50, *p* < 0.001). The relationship between MPS and PM_CSA_ for different body weights categories are shown in Fig. 5.

### Analysis of inter- and intrarater agreement

Inter- and intrarater agreement for the metric MPS and all CT-derived sarcopenia parameters were very good. The results are given in Table 3.

**Table 3:** Results of the inter- and intrarater evaluation of metric mucus plug score, muscle cross sectional area and muscle density of the pectoralis and erector spinae muscle.

		Intraclass correlation coefficient	95% confidence interval
MPS_metric_ rater 1	MPS_metric_ rater 2	0.959	0.941–0.971
MPS_metric_ rater 1	MPS_metric_ rater 1 (2^nd^ rating)	0.925	0.879–0.953
PM_CSA_ rater 1	PM_CSA_ rater 2	0.974	0.613–0.994
ESM_CSA_ rater 1	ESM_CSA_ rater 2	0.899	0.744–0.960
PM_CSA_ rater 1	PM_CSA_ rater 1 (2^nd^ rating)	0.969	0.922–0.988
ESM_CSA_ rater 1	ESM_CSA_ rater 1 (2^nd^ rating)	0.929	0.823–0.972
PM_MD_ rater 1	PM_MD_ rater 1 (2^nd^ rating)	0.955	0.883–0.983
ESM_MD_ rater 1	ESM_MD_ rater 1 (2^nd^ rating)	0.974	0.931–0.990

MPS, mucus plug score; PM, pectoralis muscle; ESM, erector spinae muscle; CSA, cross sectional area; MD, muscle density.

## Discussion

MP as well as sarcopenia are both associated with an increased risk of mortality in patients with COPD. The aim of this study was to determine whether there is a relationship between the presence of MP and CT-derived sarcopenia parameters. While PM_CSA_ and ESM_CSA_ were not significantly associated with MPS in the multivariable analysis, interaction analysis demonstrated a significant modification of the association between PM_CSA_ and MPS by body weight. In patients with a higher MPS (≥ 3) body weight and PM_CSA_ were significantly negatively correlated. To the best of our knowledge, no study investigated the relationship between these parameters in a collective of patients with advanced COPD (GOLD stage 3 and 4 disease) up to now.

Three accepted methods for quantification were used: First, the amount of mucus was quantified on CT using the MPS introduced by Dunican and coworkers. In our study 31 out of 123 patients had a MPS of greater than 0 and at most 2, 29 patients had a MPS of 3 or more, which is slightly above the proportion found in literature^5,24^.

Second, the sarcopenia parameters PM_CSA_ and ESM_CSA_ were measured. Different CT-derived parameters of sarcopenia have been introduced, including PM_CSA_ and ESM_CSA_, and their optimization is still ongoing^33–35^. The derived values for PM_CSA_ and ESM_CSA_ for patients with advanced COPD (stage 3 and 4 disease) were comparable to the values found in literature^14,36^. While a higher MPS was associated with a significantly lower body weight, there were no significant group difference in terms of PM_CSA_ and ESM_CSA_. This was an unexpected finding, given that weight loss and lower body weight are commonly associated with lower muscle CSA in both the peripheral and trunk musculature^32,37–39^. Interaction analysis revealed that the association between PM_CSA_ and MPS was modified by body weight, with a significant negative correlation between PM_CSA_ and body weight in patients with higher MPS (≥ 3). This could be of prognostic relevance, as a reduced PM_CSA_ and ESM_CSA_ are significantly associated with increased mortality in patients with COPD^40^. ESM_CSA_ showed no significant association with body weight and was not a significant predictor of MPS in multivariable analyses.

Third, PM_MD_ and ESM_MD_ as parameters of muscle composition were measured. Besides muscle quantity, muscle quality is of relevance in sarcopenia. Greater levels of inter- or intramuscular adipose tissue can negatively affect the musculature’s strength^41,42^. In our collective an increased MPS wasn’t associated with any significant difference in PM_MD_ and ESM_MD_ values suggesting an unchanged muscle composition without any deterioration in muscle quality.

There is a positive correlation between muscle CSA and muscle strength^43^. According to literature, both, a larger muscle CSA and lower amounts of intramuscular adipose tissue are associated with improved exercise capacity in patients with COPD^44,45^. Besides the decrease in muscle CSA associated with weight loss is less pronounced if the weight loss is accompanied by training and thus increased physical performance^46–48^. This suggests that patients in our cohort with a high mucus burden might partially compensate for impairments resulting from increased MPS by increased muscle quantity of the pectoral muscles while muscle quality remains unchanged.

Besides reduced muscle CSA resp. muscle mass and lower FEV1 values are associated in both healthy and diseased individuals^49–52^. Patients with a higher MPS showed significantly lower FEV1 values, similar to previous reports^24^. We hypothesize that a compensatory larger PM_CSA_ at lower weight might be an indication of the greater muscular effort required when breathing. In line with this, in the literature a higher MPS was not associated with a significantly poorer performance in the 6-min walk test^24^.

Inter- and intrarater agreement for the metric MPS were very good and within the range of the literature^29^. Muscle CSA and MD have been introduced as a reliable tool for measuring CT-derived sarcopenia parameters, which could be confirmed in our study by very good inter- and intrarater agreement^15,36^.

The following limitations have to be considered: the selection of patients was limited due to the monocentric study design and patients presented for evaluation of lung volume reduction therapy. Consequently, only patients with advanced COPD (GOLD stage 3 and 4 disease) were included. Only mucus plugs in medium to large airways were considered, as the obstruction of small airways (< 2 mm) cannot be adequately assessed by CT. The sample size was too small to form more subgroups. There was no determination of muscle strength and anthropometry or bioelectrical impedance. Only thoracic PM_CSA_ and thoracolumbal ESM_CSA_ could be evaluated on standard chest CT scans but not different visceral and subcutaneous adipose tissue compartments as in abdominal CT scans^53^. Lastly, no detailed information was available on the dietary habits of the patients.

Imaging-based evaluation of mucus plugging and parameters of sarcopenia is reliable on routine chest CT scans in patients with COPD GOLD stage 3 or 4. In our cohort, patients with more advanced COPD exhibited a higher MPS and PM_CSA_ in relation to their body weight, possibly due to the greater muscular effort required for breathing.

## Data Availability

Data generated or analyzed during the study are available from the corresponding author by reasonable request.

## Abbreviations

MP: Mucus plugging
MPS: Mucus plug score
PM: Pectoralis muscle
ESM: Erector spinae muscle
CSA: Cross-sectional area
MD: Muscle density
